# Cultural Differences in Face Recognition and Potential Underlying Mechanisms

**DOI:** 10.3389/fpsyg.2021.627026

**Published:** 2021-04-13

**Authors:** Caroline Blais, Karina J. Linnell, Serge Caparos, Amanda Estéphan

**Affiliations:** ^1^Groupe de Neurosciences Sociales, Département de Psychoéducation et de Psychologie, Université du Québec en Outaouais, Gatineau, QC, Canada; ^2^Department of Psychology, Goldsmiths University of London, London, United Kingdom; ^3^Laboratoire DysCo, Université Paris 8, Saint-Denis, France; ^4^Institut Universitaire de France, Paris, France; ^5^Département de psychologie, Université du Québec à Montréal, Montréal, QC, Canada

**Keywords:** face processing, culture, visual perception, cultural psychology, face identification

## Abstract

The ability to recognize a face is crucial for the success of social interactions. Understanding the visual processes underlying this ability has been the focus of a long tradition of research. Recent advances in the field have revealed that individuals having different cultural backgrounds differ in the type of visual information they use for face processing. However, the mechanisms that underpin these differences remain unknown. Here, we revisit recent findings highlighting group differences in face processing. Then, we integrate these results in a model of visual categorization developed in the field of psychophysics: the RAP framework. On the basis of this framework, we discuss potential mechanisms, whether face-specific or not, that may underlie cross-cultural differences in face perception.

Vision has long been considered as encapsulated, immune from higher-level influences. Because of this conception, the necessity of testing participants representing the diversity of individuals composing our world has not always been as emphasized as it is today. In the field of visual psychophysics, going back only 15 years from now, a majority of studies were based on very small and homogeneous samples, most often composed of participants of White-European descent. With the emergence of the field of cultural psychology, the reliance on homogeneous samples of participants has been questioned and even labeled as “WEIRD,” an acronym for samples composed of individuals from *Western, Educated, Industrialized, Rich*, and *Democratic* countries (Henrich et al., 2010a,b). An increasing number of studies are now revealing that individuals coming from different geographical areas or having different cultural backgrounds show differences in visual processes that have long been assumed to be universal (e.g., Segall et al., 1963; Morris and Peng, 1994; Chua et al., 2005; McKone et al., 2010).

The field of visual perception features pioneering studies in cultural psychology, amongst them studies showing that visual illusions, such as the Müller-Lyer effect, are weaker in some remote societies (Rivers, 1905; Segall et al., 1963). Since then, a large majority of the research investigating the impact of culture on visual perception has focused on comparing East-Asian and Western populations. This research has highlighted behavioral patterns suggesting that East-Asians and Westerners differ in the way they deploy their attention over the visual environment, with East-Asians spreading their attention more broadly than Westerners (Ji et al., 2000; Kitayama et al., 2003; Nisbett and Masuda, 2003; Nisbett and Miyamoto, 2005; Boduroglu et al., 2009; McKone et al., 2010). For example, when asked to identify a target letter in a hierarchical figure, like a large letter “E” (the global level) composed of smaller letters “T” (the local level), East-Asians prioritize global information more strongly than Westerners (McKone et al., 2010). In a related vein, it has been shown that East-Asians are better than Westerners at detecting changes (e.g., a square changing color, or an object disappearing) in their peripheral visual field, whereas Westerners are better than East-Asians at detecting changes in their central visual field (Masuda and Nisbett, 2006; Boduroglu et al., 2009). Finally, when viewing visual scenes during a memorization task, East-Asians fixate the background more than Westerners, whereas the latter fixate focal objects more (Nisbett and Masuda, 2003; Chua et al., 2005; Nisbett and Miyamoto, 2005).

Recent advances have also revealed the presence of important cultural variations in the core processes involved in face recognition (e.g., Blais et al., 2008; Miellet et al., 2011; Caldara, 2017; Tardif et al., 2017). The general pattern of findings with face recognition is consistent with the results, described above, typically obtained with non-face objects. More specifically, compared with Westerners, East-Asians tend to rely more on peripheral vision to process facial features, congruent with the idea that they may spread their attention more broadly on face stimuli, as was suggested for non-face objects. Given the similarity in the patterns obtained with faces and non-face objects, one may be tempted to interpret the East-vs-West differences by appealing to the same theoretical models for the two classes of stimuli. However, in the field of face recognition, many studies have provided evidence that faces should be considered a special class of stimulus, relying on specific processes that differ from non-face processes (Duchaine and Yovel, 2015; but see Gauthier and Bukach, 2007). In the present article, we will revisit the cultural differences that have been observed in face recognition. Most importantly, we will draw from theories that have been proposed in the fields of visual perception and cultural psychology to discuss potential mechanisms, whether face-specific or not, that may underlie cross-cultural differences in face perception. It is worth noting that the present article will focus on face recognition and will not cover the rich literature on how culture impacts facial expression of emotions (e.g., Yuki et al., 2007; Jack et al., 2016; Cordaro et al., 2018; Yamamoto et al., 2020). In fact, face recognition and facial expression processing rely on partly different mechanisms and cerebral pathways (Haxby et al., 2000; Kanwisher and Yovel, 2006; Duchaine and Yovel, 2015). For instance, emotional expression reflects emotional experience, which itself can vary from one culture to another (Matsumoto et al., 2008). Thus, cultural variations in facial expressions and their processing are likely to involve other mechanisms than the ones underlying face recognition, and a review and discussion of this literature would be beyond the scope of the present work.

## Face Perception Is Not Universal

The ability to recognize a face has been crucial for the survival of our species, allowing us to distinguish friends, with whom collaboration is likely, from foes who are a potential threat. A long tradition of research has investigated the visual processes underlying this ability, for instance in terms of the eye movements involved in the sampling of visual information or in terms of the nature of the information which is extracted.

The first studies that recorded eye movements during face processing revealed a triangular pattern, where fixations on the eyes and mouth areas were most frequent (e.g., Yarbus, 1965/1967). Although this average pattern has long been considered universal, studies have since highlighted the presence of important inter-individual variations (Peterson and Eckstein, 2013; Mehoudar et al., 2014). Most importantly for the present discussion, differences have been documented between cultural groups (Blais et al., 2008). Specifically, East-Asians fixate more on the center of faces and less on the eyes and mouth areas than Westerners. Interestingly, however, both groups rely on the information contained in the eyes and mouth area, as was later demonstrated using a gaze-contingent paradigm (Caldara et al., 2010): when only a small area (measuring 5 degrees of visual angle or less) around the fixation location is visible, East-Asians fixate the eyes and mouth to a similar degree as Westerners. This suggests that, under normal viewing conditions, East-Asians actually process the eyes and mouth areas while fixating on the center of faces; they thus rely more on peripheral processing than Westerners to extract facial information (Miellet et al., 2013). Taken together, these results are congruent with the aforementioned findings in hierarchical-figure and scene perception suggesting that East-Asians rely more on global visual information and peripheral processing by applying a broader spread of attention.

If the different eye fixation patterns observed for East-Asians and Westerners reflect differences in the spread of their attention then, given the links between the spread of attention and the spatial resolution of the extracted visual information (Shulman and Wilson, 1987; Balz and Hock, 1997; Goto et al., 2001), one should expect East-Asians to rely more on lower spatial frequencies than Westerners when they process faces. A study by Tardif et al. (2017) indeed found such differences in the spatial frequency tuning of East-Asians and Westerners, both when they identified faces and when they categorized them based on familiarity. Moreover, it was later shown that these differences emerge during the early stages of face processing, with East-Asians using lower spatial frequencies than Westerners as early as 30 ms after stimulus onset (Estéphan et al., 2018). This early time course implies that the differences observed are not related to late decisional processes, such as social norms dictating where to look in a face, but instead tap into early automatic processes. Such processes could potentially be bottom-up, primarily guided by information saliency; alternatively, they could be guided, in a top-down manner, by mental representations of the stimuli shaping attentional habits during stimulus processing. Taken together, these results indicate that marked differences can be found in the very nature of the visual information extracted by individuals coming from different cultures. In fact, spatial frequencies are considered amongst the most basic kind of visual information processed by the primary visual system (Tootell et al., 1981; DeValois and DeValois, 1988; Everson et al., 1998; Sowden and Schyns, 2006).

## A Framework Within Which to Think About These Cross-Cultural Differences

However interesting it is to reveal differences in visual-sampling processes between East-Asians and Westerners, as of now we do not know the mechanisms that underlie such differences. We think that the RAP framework (Gosselin and Schyns, 2002), a model of visual categorization, may offer an interesting starting point from which to explore the potential mechanisms that cause the observed differences.

The RAP framework proposes that the visual information that can be efficiently used by an observer to perform an object categorization task, called potent information, results from an interaction between the visual information available in the object which needs to be categorized and the visual information represented in the observer's memory from previous encounters with similar objects. Here, the term “object categorization task” is used in its broadest sense, referring to tasks involving the identification or categorization of a visual stimulus, for instance a face, a letter, a written word, or a visual scene. RAP is an acronym for R ⊗ A = P, where R is the visual representation of an object that is stored in memory, A is the available visual information contained in that object, ⊗ is an interaction term, and P is the potent visual information to recognize or categorize that object.

**Figure 1:**
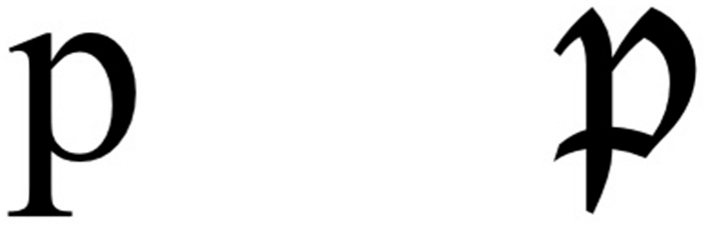
On the left side, a p written in the font Times New Roman. On the right side, a p written in the font Lucida Blackletter.

This framework entails that the potent visual information “P” will depend on the task at hand, because the available information “A” depends on the task. For instance, let us imagine the simple scenario where the task is to categorize the shape of an object that is a blue square. This object contains both color and shape information but, if one wants to categorize its shape, the fact that it is blue will not help with the decision. Thus, in the RAP framework, the available information “A” to categorize the shape of this object would be its shape, not its color. Because the potent information only includes information that is both available and stored in the visual representations, color would not be potent in this scenario. Now, let us apply this idea to a more complex stimulus and task, such as identifying the roman letter “p.” Imagine an individual who has always read texts written in the font “Times New Roman,” where the lower case “p” has the appearance depicted on the left side of Figure 1. Based on their exposure to that font, they have developed a mental representation “R” of the letter “p” containing both the curved part and the vertical tail visible in Figure 1. Now, imagine that this individual is required to read text written in the font “Lucida Blackletter.” A letter “p” written in that font is displayed on the right side of Figure 1. Notice that, in that font, an additional feature is present: a termination feature in the middle of the vertical tail, where the curved part of the “p” crosses and passes through the vertical line, creating an “x” shape. This additional feature is available “A”; it provides information that would allow an objective, computational observer to recognize the letter as a “p.” However, in the case of our individual whose mental representation of the letter “p” only includes the curved part and the vertical tail, this termination feature in the middle of the vertical tail will not be potent “P.”

According to the RAP framework, the differences reported in previous studies with regards to the spatial frequency tuning of face processing in East Asians and Westerners would be categorized as differences in potent (P) information. In fact, the method used to compare the spatial frequency tuning in both Tardif et al. (2017) and Estéphan et al. (2018), called Bubbles (Willenbockel et al., 2010; Royer et al., 2017), was shown to specifically measure potent (P) information (Gosselin and Schyns, 2002, 2004). Thus, the findings described in the previous section indicate that lower spatial frequencies are more potent for East Asians than for Westerners, and higher spatial frequencies are more potent for Westerners than for East Asians. According to the RAP framework, various factors could explain this pattern of results. First, the available information “A” may differ between Asian and Caucasian faces (for instance, because of differences in the variability of some important facial features), in a way that would predict their respective tuning. Second, even if the available information does not differ, East-Asians and Westerners may still weigh differently the importance of different kinds of facial information, and thus generate different mental representations “R.” Representational differences could emerge for multiple reasons, involving bottom-up processes such as early differences in spatial frequency sensitivity preceding face-specific mechanisms, top-down processes such as differences in attentional strategies, or both. In the next sections, we will develop these possibilities.

### Available Information and Culture

The difference in spatial frequency utilization between East-Asians and Westerners could arise from exposure to faces in which the available spatial frequencies are not the same. More specifically, if the natural variations of facial morphologies in East-Asian populations were best described by lower spatial frequencies than in Western populations, one could expect the visual system of these populations to develop visual strategies where processing lower spatial frequency information is prioritized to recognize faces.

To the best of our knowledge, available spatial frequencies have never been compared across different face ethnicities. However, the knowledge gathered so far suggests that differences in available frequencies are not to be expected. In fact, one factor that could affect spatial frequency utilization is how objectively similar the faces are within a given population. In a study by Tardif et al. (2017), it was shown that more similar faces were associated with the utilization of higher spatial frequencies in a face recognition task. Thus, differences in the available spatial frequencies could emerge if the degree of dissimilarity, or visual heterogeneity, were larger in one population than the other. However, Caldara and Abdi (2006) showed, using an image set composed of over 300 White Caucasian and East Asian faces, a similar degree of visual heterogeneity with both face ethnicities. This finding is also in line with an anthropometric study showing no evidence of differences in facial heterogeneity across three ethnic groups, namely Whites, Blacks and Asians (Goldstein, 1979).

Despite evidence pointing toward an overall similar level of heterogeneity in White and East Asian faces, the possibility remains that the level of heterogeneity of local features might differ. For instance, exemplars within one face ethnicity may vary more in terms of the shape of the mouth, whereas they may vary more in terms of the shape of the eyes within another ethnicity. Such differences could in turn affect spatial frequency tuning. Thus, we think that future studies should empirically compare available spatial frequencies to allow a better understanding of the mechanisms underlying cultural differences in face recognition.

### Visual Representations and Culture

Another factor potentially explaining cultural differences in face recognition is that East-Asians and Westerners may weigh spatial frequencies differently when generating representations of faces that are then stored in memory. But why would this happen? One possibility is that a generally higher sensitivity to lower spatial frequencies (or higher spatial frequencies) could translate, via bottom-up processes, into the creation of visual representations of faces tuned toward lower (or higher) spatial frequencies. This possibility has been evaluated as a first candidate mechanism in a study by Tardif et al. (2017). The contrast sensitivity function was measured in two separate tasks using non-face stimuli (sinusoidal gratings) and compared between East Asian and Western participants. No difference was found, suggesting that the difference observed with faces is not caused by differences in early sensitivity as such (Tardif et al., 2017).

Differences in visual representations could also emerge because, as the visual system develops, various factors modulate the attentional processes involved in the viewing of complex objects such as faces. The few hypotheses that have been proposed so far to explain the cultural differences in visual perception, which we will describe in the next paragraphs, all posit the existence of factors that bring about differences in the way attention is deployed during the processing of visual objects and/or faces. Such differences affect the input received by the visual system: when deploying attention over a narrower spatial area, the spatial resolution of the processed visual information becomes higher (Balz and Hock, 1997; Goto et al., 2001). Thus, if the visual input received by observers with different attentional strategies differs, the visual representations (R) built upon that input will likely differ. Down the line, these different representations (R) would lead to differences in potent (P) information, even when the available (A) information is controlled, for example, in lab settings.

One theory in the field of cultural psychology posits that exposure to an individualistic vs. collectivistic system of values impacts general perception in a way that could be congruent with the pattern of results found with faces (Nisbett et al., 2001). More specifically, this theory proposes that individuals exposed to individualistic systems of values perceive the world in a more analytical manner, for instance by narrowing their attention, which would facilitate the processing of focal objects. In contrast, individuals exposed to a more collectivistic system of values would perceive the world in a more holistic manner, by spreading their attention more broadly and processing the context more. This theory is supported by many visual-perception studies investigating differences between East-Asian and Western individuals with non-face objects (Ji et al., 2000; Kitayama et al., 2003; Nisbett and Masuda, 2003; Nisbett and Miyamoto, 2005; Boduroglu et al., 2009; McKone et al., 2010). Under this theoretical framework, exposure to collectivistic (vs. individualistic) values could drive individuals to deploy their attention more broadly (vs. less broadly) over faces, leading to different representations (R). These representations would, in turn, bring about the observed differences in potent (P) information, whereby East Asians and Westerners rely on different spatial frequencies and eye movement strategies during face recognition. However, the evidence that cultural differences in face processing can be explained by differences in individualism-collectivism is tenuous at best (e.g., Kelly et al., 2011; Liu et al., 2019).

More recently, additional findings have given rise to an alternative hypothesis to explain general perceptual differences between individuals coming from different cultures. In particular, traditional Himba individuals - a population coming from a remote part of northern Namibia - display behavioral patterns congruent with a narrow spread of attention and a reliance on analytical processing, despite living in a more interdependent society than Western individuals (Caparos et al., 2012). For instance, they are less affected by the Ebbinghaus illusion, suggesting that they can more easily ignore the context in which an object appears, when the task requires to do so. Interestingly, a series of studies show that behavioral patterns are congruent with the spread of attention increasing with urban exposure (Caparos et al., 2012, 2020; Linnell et al., 2013; Bremner et al., 2016).

Specifically, in a recent paper, two of the present authors (Linnell and Caparos, 2020) proposed that urban exposure promotes changes in the neuromodulatory locus coeruleus-norepinephrine (LC-NE) arousal system, and this results in the adoption of an explorative mode of visual sampling. They proposed that this shift may impact both covert and overt attention (where covert attention involves attending without moving the eyes whereas overt attention involves eye movements toward the attended location). According to this view, an increased arousal state associated with urban exposure could lead both to covertly attending to broader areas of space and to increasing overt spatial exploration through more eye movements toward non-focal objects. Thus, according to this theoretical framework, an increased arousal state could impact attention distribution and lead to the development of face representations (R) that are in lower spatial frequencies, thereby leading to differences in potent (P) information. Nevertheless, this hypothesis remains speculative and several other factors could explain the differences observed between rural and urban populations with non-social visual stimuli. With regard to face stimuli, the studies comparing East Asians and Westerners have not controlled for the degree to which participants had been exposed to urban environments. In many eye-tracking studies (e.g., Blais et al., 2008; Caldara et al., 2010; Rodger et al., 2010; Miellet et al., 2013), all of the participants were tested in the same city (Glasgow, Scotland) but could have grown-up in any village or city of a Western or East Asian country. In other eye-tracking studies (e.g., Miellet et al., 2010, 2012; Kelly et al., 2011) as well as studies comparing spatial frequency tuning for faces (Tardif et al., 2017; Estéphan et al., 2018), Western participants were tested in a medium sized city (Gatineau, Canada or Glasgow, Scotland; populations of ~280 and ~600 K, respectively) and East Asian participants were tested in a large city (Hangzhou or Guangzhou, China; populations of ~10.3 million and ~15.3 million, respectively), but again they could have grown-up in any village or city of a Western or East Asian country. Thus, in all of these studies, the main variables associated with the two aforementioned theories, that is, urban exposure and exposure to an individualistic vs. collectivistic system of values, were confounded. Thus, further studies examining face processing in populations within the same culture but with varying degrees of urban exposure would help disentangle the two potential explanations described above, namely exposure to different systems of values and exposure in different degrees to an urban environment.

Moreover, both of these potential explanations make the prediction that cultural difference in the spatial frequency sensitivity function and eye movements in face processing generalizes to other classes of objects than faces. For instance, cultural differences observed in eye movements during face processing have been shown to generalize to homogeneous non face objects (Kelly et al., 2010). In addition, previous neuroimaging findings have shown a cultural specialization, during non-face object processing, in areas of the visual cortex associated with the processing of spatial frequencies (Ksander et al., 2018). However, the spatial frequency content of the stimuli was not manipulated during the experiment – only broadband stimuli were presented – and the interpretation that the cultural difference in terms of cerebral activity reflected the processing of different spatial frequencies was made using a posteriori analyses. In contrast, Tardif et al. (2017) found no difference in spatial frequency tuning between East Asians and Westerners when using low-level sinusoidal gratings. Likewise, studies on letter identification with spatial frequency manipulation might point toward non-generalizability of cultural differences, at least where letters are concerned. Like faces, letters (or characters) are prevalent in many countries and represent a culturally meaningful visual input. Interestingly, it has been demonstrated that spatial frequency use for letter identification is determined by letter stroke frequency (Majaj et al., 2002) or letter complexity (Wang and Legge, 2018), where more complex characters require higher spatial frequencies for reliable recognition. Chinese characters typically contain higher stroke frequencies/more complexity than common alphabetical letters (e.g., Wang et al., 2014). If letter complexity is a determining factor for spatial frequency use, we might for instance expect Chinese observers to use higher spatial frequencies than Western observers during reading. However, this idea remains speculative since, to our knowledge, Chinese observers have not been directly compared with Western observers on such tasks.

These results pertaining to spatial frequencies with non-social stimuli are very interesting, with some pointing toward a possible generalization of cultural differences in spatial frequency tuning across different stimulus classes (e.g., Kelly et al., 2010; Ksander et al., 2018) and some not (e.g., Majaj et al., 2002; Tardif et al., 2017; Wang and Legge, 2018). A possibility worth considering is that cultural differences in attentional deployment might interact with the nature of the task and stimuli. For instance, it is possible that the range of available spatial frequencies is wider for faces and homogeneous objects, thus allowing an observer to select information in accordance with their “default” attentional bias: higher spatial frequencies for Westerners and lower spatial frequencies for East Asians. However, with other classes of stimuli, such as sinusoidal gratings and perhaps letters, the available information might be more constrained, thus forcing individuals from both cultural groups to rely on the same range of spatial frequencies. To properly address the question of generalizability, more studies using an experimental paradigm designed to measure the impact of culture on the utilization of spatial frequencies with non-face objects are still needed. Those studies should also manipulate the range of available spatial frequencies of the stimuli in order to explore the possible interaction between attentional biases to a range of spatial frequencies and the nature of the visual input.

In fact, if cultural differences in the spatial frequency sensitivity function in face processing do not generalize to other classes of objects than faces, then this may indicate that face-specific mechanisms underpin this pattern of finding. As explained in the Introduction section, multiple studies point to the existence of face-specific mechanisms. It is true that the pattern of findings with faces integrates well within the general framework where East-Asians, or individuals leaving in highly urbanized environments, are more global and/or spread their attention more broadly, whereas Westerners, or individuals living in less urbanized environments, are less global/more local and/or spread their attention more narrowly. However, it remains possible that this is either just a coincidence or only part of the explanation.

If the difference in the spatial frequency sensitivity function is face-specific, it could emerge from social practices promoting the encoding of specifically face representations using different spatial resolutions. One potential mechanism that we believe could have a face-specific impact is the spontaneous distance occurring between a mother's face and her infant's eyes. In fact, as distance increases between a stimulus and an observer, the availability of higher spatial frequencies decreases. To the best of our knowledge, only one study has compared mother-infant distance during face-to-face interactions in East-Asian and Western populations (Fogel et al., 1988). This study revealed that Japanese mothers stand farther from their child compared with American mothers. Thus, by affecting the visual information to which babies have access (the available (A) information), the mother-infant distance could in turn promote the encoding of visual representations (R) of faces in lower spatial frequencies in East Asians than in Westerners, thereby leading to the observed differences in potent (P) information. Given that faces are the most frequent stimuli to which an infant is exposed (Sinha et al., 2007), cross-cultural differences in mother-infant distance during early development may be an important factor to consider when attempting to understand the representation of visual faces across cultures.

## Conclusions and Future Directions

The ability to process faces is of the utmost importance for the success of our social interactions. Yet, it has now become clear that individuals can achieve similar abilities at this task while using strikingly different strategies of visual-information sampling. We started this article by alluding to the idea of encapsulated vision: could the East-West differences in low-level face processing be considered evidence against the theory that vision is modular (Pylyshyn, 1999) and that perception is not influenced by cognition (Firestone and Scholl, 2016)? We do not think this is the case. In fact, according to Firestone and Scholl, in order to qualify as evidence of top-down influence of cognition on perception, an effect must not be explained by differences in attentional strategies, since attention affects the input received by the visual system. As discussed throughout the present article, East-West differences in the visual processes underlying face recognition most likely reflect differences in the way these groups of individuals deploy their attention over space. Moreover, although the mechanisms underlying the development of such differences in attentional deployment remain unknown, the plausible candidates discussed here all represent long-term influences shaping visual information extraction processes: being exposed to individualistic or collectivistic systems of values, modulation of arousal associated with different degrees of urban exposure, or a face-specific mechanism emerging from differences in social norms for interpersonal distancing. These long-term influences may in fact shape visual processes such that the differences observed in adults of different cultural groups are bottom-up rather than top-down. The finding, by Estéphan et al. (2018), that differences in spatial frequency tuning across East Asians and Westerners emerge as early as 30 ms following stimulus onset supports this idea. More research will be needed to understand the mechanisms underlying the cultural differences observed in visual perception. Such an understanding will in fact be needed to decide whether these cross-cultural differences in face processing can be considered evidence against the idea of encapsulated vision.

We believe that the ideas presented above emphasize the importance of including more varied participant samples - rural, urbanized, and exposed to different systems of values and social norms - as well as different object classes - faces compared to complex scenes or simple objects or characters - in order to better understand the visual mechanisms that are specific or not specific, as the case may be, to face perception.

Moreover, when comparing populations with different cultural backgrounds, one needs to take great care to ensure that the methods used are comparable, for instance, as regards the participants' familiarity with the stimuli presented, or how they understand instructions and tasks. Methods involving implicit measures, in which responses are not required by the participants, may be ideally suited to working around problems with instructions and tasks. One potentially interesting paradigm for addressing the question of attentional breadth and spatial frequency tuning is pupillometry. In fact, studies have shown that pupil dilation is associated with both increasing attentional breadth (Daniels et al., 2012) and the processing of lower spatial frequencies (Hu et al., 2019).

In summary, the task of untangling the mechanisms that underpin face recognition is an intricate one. The development of face perception remains nested in multifaceted cultural backgrounds that we can only ever approximate with current measures. Following up on this line of thought, it becomes ever more apparent how necessary it is to explore the interaction between culture and face perception, and then from diverse perspectives.

## Author Contributions

CB wrote the first manuscript draft. CB, KL, SC, and AE contributed to revisions of the following drafts and final version of manuscript. All authors contributed to the article and approved the submitted version.

## Conflict of Interest

The authors declare that the research was conducted in the absence of any commercial or financial relationships that could be construed as a potential conflict of interest.
